# Integrated Care for Older People in France in 2020: Findings, Challenges, and Prospects

**DOI:** 10.5334/ijic.5643

**Published:** 2021-11-08

**Authors:** Emma Bajeux, Aline Corvol, Dominique Somme

**Affiliations:** 1 Department of Epidemiology and Public Health, Rennes CHU, FR; 2 Department of Geriatrics, Rennes CHU – Univ Rennes, CNRS, Arènes-UMR 6051, F-35000, Rennes, FR

**Keywords:** France, health and social integration, gerontology, older people

## Abstract

**Background:**

We analyze here major changes over the last decade in the French healthcare system for older people, in terms of the integrated care concept.

**Policies:**

During this period, the major theme of public policies was “care coordination.” Despite some improvements, the multiplication of experimental programs and the multiplicity of stakeholders distanced the French healthcare system from an integrated care model. Professionals and organizations generally welcomed these numerous programs. However, most often, the programs were insufficiently implemented or articulated, notably at a clinical level, because of the persistence of a high level of fragmentation of governance, despite the creation of regional health agencies 10 years ago. The COVID-19 crisis has highlighted this fragmentation. Moreover, we still lack data on the impact of these programs on people’s health trajectories and personal experiences.

**Conclusion:**

The French healthcare system seems more fragmented in 2020 than in 2010, despite improvements in the culture of professional collaboration. The future health reform is an opportunity to capitalize upon this progress and to implement “integrated care.” This implies a strong and continuous national leadership in governance and change management.

## Introduction

In January 2020, over one-quarter of the French population was aged 60 or more [1]. As in all developed countries, the number of older people had been increasing for over 30 years, with a spurt since the mid-2010’s with the arrival of the so-called baby boom generation [2]. About 1,459,000 people over 60 years of age living at home are currently deemed to be dependent, to which should be added 584,000 people living in institutions, meaning that there are just over 2 million dependent older people in France [3]. Projections indicate that this number will increase by 200,000 to 410,000 (+15% to +33%) between 2015 and 2030 [2]. France could have around 4 million senior citizens with a loss of autonomy in 2050, representing 16.4% of people aged 60 or over (compared to 15.3% in 2015) [2].

In France, as in other European countries, spending on the care of older people is increasing. All payers considered, it was 30.0 billion€ in 2014, i.e. 1.40% of the gross domestic product (GDP). Over three-quarters of this amount is provided by the public authorities. Public spending on dependent older people is anticipated to rise by 2060 to 2.07% of GDP, and total spending will almost double to 2.78% of GDP [4].

In this policy paper, we define integration, according to the definition of the French Society of Geriatrics and Gerontology, as a process designed to overcome the fragmentation of services for vulnerable people, requiring an inter-sectoral and multilevel approach, and, according to the definition of Kodner and Kyriacou, as a set of techniques and organizational models designed to create connectivity, alignment, and collaboration within and between the treatment and care sectors at the funding, administrative, and/or provider levels [5,6]. The goals are to enhance quality of care and quality of life, patient satisfaction, and system efficiency for patients with complex problems cutting across multiple sectors and providers [7]. Fragmentation is apparent as “lack of coordination between the different levels and settings of care, duplication of services and infrastructure, unutilized productive capacity, and health care provided at the least appropriate location, especially hospitals.” It impairs users’ access to services and continuity of care [8].

Ten years ago, in spite of attempts to improve their articulation and coordination, French health and social services appeared fragmented [9]. Regarding governance, the regional level had been strengthened and simplified by the creation of regional health agencies that manage both the financing and planning of healthcare, as well as medical-social care and services and the supervision of health policies for older people. New innovative organizations called MAIA,^1^ i.e. “Method of Action for Integration of Health and Social Care in the field of Autonomy,” were implemented as the first real model of integration services in France involving all stakeholders in the medical, psychological, social, administrative, and environmental fields, at all levels of responsibility, from national to local and clinical. However, the construction of policies for older people remained the responsibility of several bodies, while the need for national political leadership appeared central to the promotion of integrated care.

The aim of this policy paper is to describe the evolution over the last 10 years of key policies and organizations promoting care integration for older people in France, by updating published data [9], so as to identify barriers and facilitators in the implementation of integrated care and to highlight the main issues and challenges.

Due to the scarcity of relevant published data, the findings described here largely rely on the field experience of the three authors, who are physicians and researchers in geriatrics and public health.

## Description of policy development, implementation, and evaluation

### National level

At the national level, French health policies on care and services for older people are as fragmented as they were 10 years ago. At the Ministry of Health and Solidarity, healthcare and social services are planned in one department (Directorate General of Healthcare Provision), while public policies for solidarity and the promotion of equality, to foster social cohesion and support for people’s autonomy, are planned in another department (Directorate General of Social Cohesion). Thus, the National Solidarity Fund for Autonomy, under the supervision of the Directorate General of Social Cohesion, is responsible for funding assistance to older people with loss of autonomy, and also provides expertise and information, and hosts events for this public, but has limited responsibility for managing the provision of health and medical-social care.

Over the last decade, the laws defining health policies concerning older people also reveal a lack of cohesion. In 2015, Parliament enacted a law for the adaptation of society to “population aging” [10]. One of its main thrusts concerned an increase in the independence social allowance to combat inequality, and the introduction of a new tax to fund prevention and support regarding loss of autonomy. Significantly, this text does not mention the notions of “integrated care and services” and “integration.” The law on modernization of the health system, promulgated only one year later, defined new healthcare organization on a local level (described below) by emphasizing the development and structuring of primary care teams, which became the linchpin of the patient pathway in the health, social, and medical-social sectors [11]. The parallel and independent drawing up and promulgation of these two health laws, which modify older people’s care organization and pathways, appear to reflect a lack of integration of social and healthcare questions in political planning in France.

Moreover, there is currently no annual, integrated, and complete vision of public expenditure for older people. In fact, medical-social expenditure specific to older people is identified in a subset of the annual global budget for French National Health Insurance (Objectif National des Dépenses d’Assurance Maladie), while expenses related to hospital or outpatient care are included in dedicated budgets and those related to older people cannot be individualized. In 2020, French National Health Insurance was set at 205.3 billion€, of which 45.5% was for outpatient care, 41.0% for healthcare institutions, and 4.9% specifically for institutions and services for older people. In addition to French National Health Insurance, other sources of funding are dedicated to dependence, including national taxes on professional activities.

### Regional and local levels

Local management of policies regarding older people remains complex in France because both territorial councils,^2^ which are defined by law as social action leaders, and regional health agencies are all involved in organization of the health system. National health policies concerning older people are passed on and adapted to regional health priorities in a program drawn up and led by the regional health agencies created over 10 years ago [12]. The role of elected departmental councils is central to planning the provision of social and medical-social services (agreement and price setting for home care services, retirement homes…). These councils are also responsible for organizations like local information and coordination centers and for allocation of the home care allowance for the older people. Given this situation, territorial meetings of funding bodies were proposed in the framework of the 2015 law [10], so as to define a one-stop approach and a joint strategy for funding and support services for older people. However, in the end, this mission was often restricted to the prevention of loss of independence of older people, which may result in a new line of fragmentation between prevention and care.

Locally, hospital groups (Groupements Hospitaliers de Territoire) were created in 2016 throughout France [13], with a view to improving coordination between public hospital care providers. These hospital groups were centered on a support facility for a defined territory so as to facilitate joint and graduated patient care in intra-hospital care systems, formalized in a joint medical and nursing project. The aim was to switch from a “facility-centered approach” to a “patient-centered approach.” However, in addition to excluding private establishments, the hospital groups have hitherto had few links with primary care physicians and specialists operating in private practice, the social sector, or the medical-social sector, and most often have failed to link hospitals into mental health networks.

One perspective for greater integration within hospital groups is the recently proposed creation of “Territorial Professional Health Communities” (CPTS; Communautés Professionnelles Territoriales de Santé), which could foster an integrated conception of the provision of care and services to the population [11,14]. On their initiative, these CPTS bring together several primary care stakeholders, together with medical-social and social stakeholders, as well as professionals working in healthcare establishments in the same territory. The aim is greater coordination between these professionals, to facilitate their response to any health problems they identify, by proposing formalized objectives in a health project approved by the regional health agency through contractualization. The objective then is to move from a “medical practice population” approach to a “population” approach. These CPTS are currently being deployed in France (about 500 in the summer of 2020, of the 1000 planned by the government by 2022), and their effectiveness and impact in terms of health quality will have to be assessed. In current regulatory texts, these CPTS have few responsibilities in terms of planning the provision of care and services for older people (exclusive role of the regional health agencies and departmental councils). Furthermore, it already appears that the setting up of these CPTS varies greatly between territories, and is probably easier in zones where fewer stakeholders are involved in care provision, notably in healthcare establishments, and doubtless more complex around large hospital and university centers, because more stakeholders are concerned and because of persistent difficulties of collaboration between healthcare professionals in general practice (mainly self-employed) and in hospitals (principally salaried employees).

At the local scale, groups of self-employed healthcare professionals have since 2007 been deployed in care homes (Maisons de Santé Pluri-professionnelles). These teams of primary care professionals (at least three general practitioners and one paramedic, such as a nurse, physical therapist, dietitian…) work at the same site and draw up a health project to coordinate their actions within a defined territory. These care homes can be part of the CPTS and are funded by the regional health agencies, following approval of a healthcare project that meets the needs of the territory’s population. They serve both to fight against the desertification of care provision and to build the interprofessional collaboration needed to ameliorate the quality of care [15].

## Organizations facilitating coordination and integration

There are currently many organizations in France that are “supposed” to provide integrated care for dependent older people. They comprise experimental and regulatory organizations that sometimes overlap. Progressively, efforts are being made to move from organizations that “coordinate” to those that integrate provision of care and services. However, the real-life role of these new organizations is sometimes far from the ideal of integration. Several difficulties have arisen during the deployment of these organizations, leading to the creation of new ones (without removing the initial ones) and an attendant lack of clarity for healthcare professionals and users alike. These multiple programs were generally welcomed by professionals and organizations, but we still lack data on their impact on older people’s health pathways and experience thereof [3].

The local information and coordination centers, the gerontological health networks, and MAIA described 10 years ago still exist in the French health system, but are gradually being replaced [9]. Case management by MAIA has been implemented in most territories in France, leading to benefits for patients and their relatives [16,17]. However, some key elements of the MAIA program were progressively abandoned (joint territorial information system and joint multidimensional assessment tool). Strategic collaboration intended for decision makers and funders to take decisions locally through shared responsibility for improving care provision to the older and for fostering the process of integration was in large part pooled with other collaborative schemes, non-uniformly from one territory to another. Tactical collaboration connected operators responsible for aid and care services for the older people, albeit once again with between-territory heterogeneity. These difficulties are in part explained by national questioning of the legitimacy of the MAIA pilot schemes, which were tasked with overseeing collaborations, albeit with a limited role in planning care provision to the older people and focused mainly on the social sector [18,19].

Over the last decade, new organizations set up to promote integration share a set of common principles, i.e. an integrated entry point, defined admission criteria, and a case management process with support from multidisciplinary teams responding to the needs of patients in different areas of their lives.

A first experimental scheme for the older people at risk of loss of autonomy (Personnes Agées à Risque de Perte d’Autonomie) was initiated in 2014. This was a new method of organizing care intended to optimize the care pathway of the over-75s at risk of losing their autonomy, so as to improve their quality of life and that of their carers, to identify situations involving a risk of loss of autonomy, and to prevent gaps in the care pathway (by limiting recourse to hospitalization). The aim was to use various organizational tools to improve coordination between stakeholders in the private, hospital, and medical-social sectors. In particular, schemes for the territorial coordination of support (Coordinations Territoriales d’Appui) were created, based on existing systems of coordination and integration. These constitute an all-in-one organization designed a) to inform and guide professionals to the appropriate health, social, and medical-social resources of the territory, b) to implement social measures, where necessary, and c) to support the organization of the complex care pathways of the older people by means of case management and subsidiarity, with multidimensional assessment. The 2019 evaluation yielded interesting results and led to a change in behavior in some territories, but this was insufficient to create a real territorial dynamic [3]. In 2016, the creation of “Territorial Support Platforms” (Plate-forme Territoriales d’Appui) embodied the principle of territorial coordination of support by promoting a bottom-up approach, with emphasis on the initiatives of stakeholders on the ground, the regional health agencies acting as facilitators and regulators through contractualization [20]. While territorial coordination of support targeted only older people at risk of loss of autonomy and was requested by primary care physicians, intervention of the territorial support platforms could be triggered by users, their relatives, or healthcare professionals, unrestricted by inclusion criteria. The territorial coordination of support and the territorial support platforms thus offered support to professionals (principally first responders) in the organization of care pathways, by decompartmentalizing the various professional organizations, notably by means of a network of expertise and a shared information system.

Recently, these mechanisms (gerontological health networks, MAIA, “Territorial Support Platforms”, and territorial coordination of support for the experimental scheme for the older people at risk of loss of autonomy) have been merged into “Support Schemes for the Population and for Healthcare Professionals in Coordinating Complex Care Pathways” (Dispositifs d’appui à la population et aux professionnels pour la coordination des parcours de santé complexes) [14]. The missions of these new support schemes are broadly superimposable on those of the territorial support platforms and are designed to build close links with the CPTS.

The many changes the French health system has undergone in the last decade are hard to summarize. They include integration of care provision that is less direct but real enough, such as the development of mobile geriatric teams, the appearance since 2018 of advanced practice nurses, changes in the roles of clinical pharmacists, the possibility of merging some services, and the legal framework concerning information sharing [10,14].

**Figure 1:**
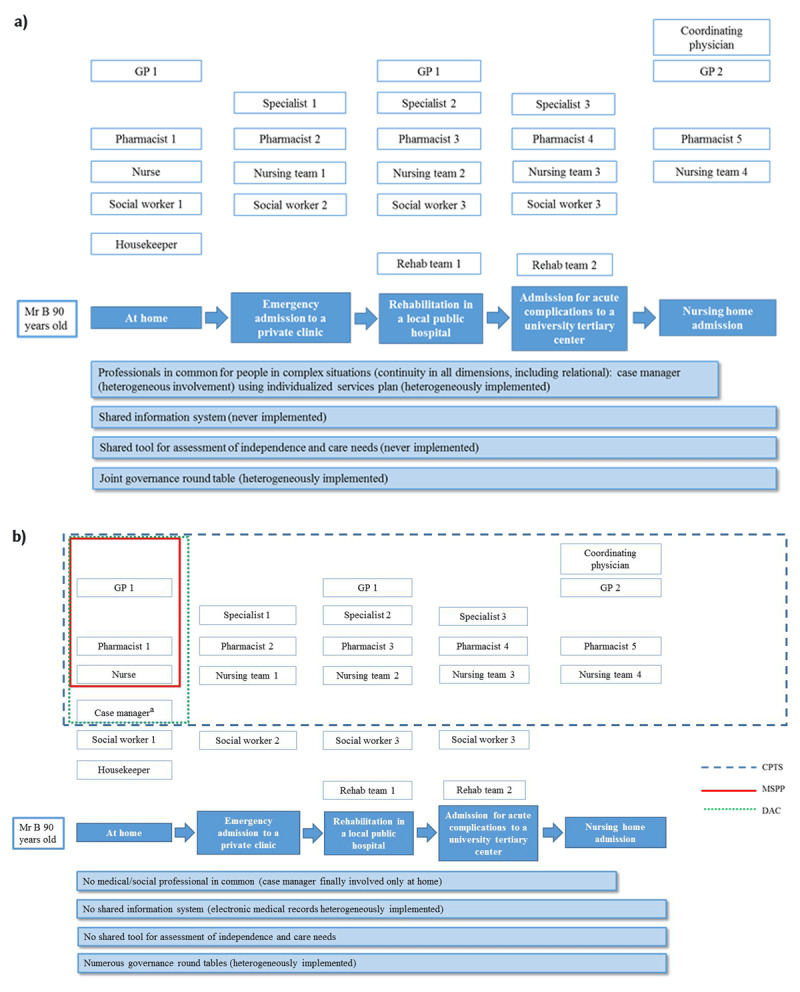
**Example of the care pathway of a 90-year-old in the French health system**. Mr B is 90 years old. He lives alone at home with help from health professionals, but without family support, and is financially vulnerable. He suffers from severe osteoarthritis and from the sequelae of previous osteoporotic fractures. His mobility is very limited and he needs help in the activities of daily living. He also suffers from mild to moderate cognitive impairment and from macular degeneration. **a) Example of the care pathway of a 90-year-old in the French health system in 2010.** In 2010, health professionals providing home care had little opportunity to collaborate and depended mostly on their regional health authority (regulated by the public health code) for the regulation of their activity. Social workers and housekeepers were mainly under the governance of the Territorial Authority (regulated by the Social Action and Family Code). Following emergency admission to hospital because of organ failure, the patient was in need of rehabilitation and, according to availability and the patient’s choice, was transferred to a private clinic near his home where his own general practitioner could attend to him. Following a subsequent complication, the patient was transferred to a university hospital, because there was no bed available at the previous clinic. Finally, the outcome was unfavorable and admission to a nursing home was considered. In this 2010 scenario, there was no continuity between the first-line medical team and the private, local, university hospital, and nursing home health and social professionals. In 2010, the policy concerning homes for the integration and autonomy of Alzheimer patients (MAIA) was being implemented, while introducing a shared information system (with training workshops during 2010), a joint assessment tool (idem), and the appointment to a new post of a supervisor who intervened wherever needed in the patient’s health trajectory so as to coordinate actions and respond to the patient’s priorities and care needs. Case managers can report to the supervisor (see text) if there is frequent or problematic fragmentation between the various organizations, and together they can report difficulties in health and social issues at strategic round tables led by the regional health authority. **b) Example of the care pathway of a 90-year-old in the French health system in 2020**. In 2020, several mechanisms are being implemented to facilitate collaboration between the various health professionals of a given territory (multidisciplinary care homes, territorial professional health communities, and territorial coordination of support for the experimental scheme for the older people at risk of loss of autonomy). However, there are no training workshops for a shared information system or for joint professional and common assessment tools, and although there is some connection between the health and social authorities, there is no systematic round table discussion. ^a^ The term “case manager” is used here to describe the professional function, but the name may vary.

***Figure 1*** illustrates the care pathway of a 90-year-old in the French health system of 2010, according to the logic of the article published 10 years ago, and as it could exist now in 2020, according to the logic of current regulatory texts, without taking into account the current highly heterogeneous implementation.

***Table 1*** summarizes the authors’ analysis of the evolution of integrated care in France between 2010 to 2020, according to a framework derived from a model described by Leutz and by Kodner et al. [7,21].

**Table 1:** Evolution of integrated care in France between 2010 to 2020 according to a framework derived from a model described by Leutz and by Kodner et al. [7,21].


FACTORS	WHAT WAS THE SITUATION IN 2010?	WHAT WAS EXPECTED FOR 2020?	WHAT HAS BEEN IMPLEMENTED IN 2020?	WHAT IS EXPECTED IN THE NEAR FUTURE?

**Funding mechanisms**	Multiple sources of funding of health expenditures for older people Payment of healthcare provider on a fee-for-services basis	Pool funds to have a complete and transparent vision of health expenditures for older people Diversification of payment of care providers to encourage pathway coordination for older people	(–) No annual integrated complete vision of health expenditures for older people until 2020 (+) Introduction of capitation payment for GPs and quality-based payment for GPs and hospital	Implementation of a fifth “autonomy” branch of the social security system to cover all provisions of care and services relating to loss of autonomy in 2021 Development of innovative funding for care providers (development of financial incentives to quality of care for care providers, funding for care pathway)

**Governance and management**	National governance fragmented in the French Solidarity and Health Ministry Regional and local governance fragmented between regional health agencies and territorial councils	Integrated governance of health policies for older people from national to local level with strong national leadership	(–) Persistence of fragmentation at the national and local levels (–) Absence of integrated care concept references in regulations and laws (+) Implementation of health care democracy	Growing implementation of health care democracy

**Jurisdictional boundaries and information-sharing**	No legal framework to share information between care and social professionals	Creation of a new legal framework to allow sharing of files Joint record for social and health providers	(+) Lifting of the restrictions on information by the 2015 law on the adaptation of society to aging (–) No joint record for social and health providers	Repeal of the law on the adaptation of society to aging scheduled for 2022

**Quality of care and system outcomes**	No systematic assessment of health policies for older people	Implementation of a clinical assessment tool allowing reporting of information	(+) Development of the consideration of patient-reported experience and outcome measures to improve the quality of care (–) No systematic assessment of health policies for older people	Development of the consideration of patient-reported experience and outcome measures to improve the quality of care Development of innovative funding for care providers (development of financial incentives to quality of care for care providers, funding for care pathway)

**Degree to which health and social care professionals collaborate, teamwork and continuity of care**	Fragmentation between health, social, and medical-social sectors Gap between hospital and private practice in primary care	Shared responsibility for health status of the older people in a geographic area Shared responsibility for financial management for health and social care for older people in a geographic area	(+) Implementation of new care organizations: teams of primary care professionals (MSP), Territorial Professional Health Communities (CPTS) including health and social care providers from hospitals and private practice, Support Schemes for the Population and for Healthcare Professionals (DAC) facilitating integration in a geographic area (–) No shared responsibility for health or financial management for the health and social care of older people in a geographic area	Implementation by CPTS of a population-based approach

**Patient screening** **and multidisciplinary assessment**	No shared tool for patient screening and multidisciplinary assessment of loss of autonomy	Common tool sharing with all health and social care provider for loss of autonomy assessment	(–) No national and shared tool for loss of autonomy assessment	No plan for a shared assessment tool

**Care management**	No case management system addressed to older people	An effective case management for older people in complex situations performed by the MAIA	(+) Implementation of case management with the deployment of MAIA (–) Lack of legitimacy of case managers due to persistent fragmentation (case managers have no delegation of power to initiate services that seem necessary) (+) Multiple innovative organization to optimize older people’s life pathway	Structuring and coherence of the multiple innovative organization to optimize older people’s life pathway


CPTS: Territorial Professional Health Communities called in French “Communautés Professionnelles Territoriales de Santé.” MSP: Group of self-employed healthcare professionals called in French “Maisons de Santé Pluri-professionnelles.” MAIA: Method of Action for Integration of Health and Social Care in the Field of Autonomy, called in French “Méthode d’Action pour l’Intégration des services d’aides et de soins dans le champ de l’Autonomie.” DAC: Support schemes for the population and for healthcare professionals in coordinating complex care pathways called in French “Dispositifs d’Appui à la Coordination et aux professionnels pour la coordination des parcours de santé complexes.” GP: general practitioner.

## Perspectives

Analyzing the benefits and limitations, for patients, carers, healthcare professionals and society, of the changes that have occurred over the past 10 years is made difficult by the scarcity of scientific data published in France evaluating both health policies and organizations implemented. To our knowledge, there is little evidence-based data to document the failure of the expected reforms in France. However, a recent study of health systems in high-income countries shows that France not only had the most gaps in hospital discharge planning in 2017 (60% versus 39% study average), but also the worst trend between 2010 and 2017 (+6% versus –10% study average). Experience of care coordination problems were reported by 31% of survey respondents (versus 27% study average), with a change of +24% (study average +0.8%) [22].

Work prefiguring draft legislation on autonomy scheduled for 2021 is underway in the follow-up to a 2018 consultation on old age and autonomy [3]. The objective is to end a system of siloed support and care of older people and to rethink support for older people in terms of a new paradigm of commitment by all healthcare, social, and medical-social stakeholders through decompartmentalization of their interventions. Faced with a lack of clarity regarding the funding of care and services for older people, consideration has been given to the creation of a fifth “autonomy” branch of the social security system (in addition to the existing branches of family, illness, workplace accidents and occupational diseases, and retirement/old age). This new fifth branch would cover all provision of care and services relating to loss of autonomy, whether for older people or for people with disabilities. The purpose would be to recognize loss of autonomy as a social protection risk in its own right, and to determine an organization and governance dedicated to protection against this risk, by improving visibility concerning its budgets and through debates on public funding of autonomy. Although the creation of this fifth branch of the social security system appears highly desirable in improving the funding of home-based social needs, the choice of management distinct from that of health insurance, by two different bodies, raises a new risk of fragmentation of the system.

Moreover, the current local systems of coordination and integration (MAIA, “Territorial Support Platforms”, “Support Schemes for Population for the Coordination of Complex Care Pathways”…) are too often poorly identified by users and healthcare professionals and are considered to be too numerous and heterogeneous. Deliberations are underway to replace these local systems by a nationwide network of homes for seniors and their carers. This all-in-one organization would constitute a nexus of information, guidance, support, and explanation of rights. It would also jump start the coordination of social, medical-social, and health interventions for the older people and their carers, in particular for more complex support, and would be led by the National Solidarity Fund for Autonomy. Joint management of homes for seniors and carers would be set up between regional health agencies and departmental councils to guarantee integration of healthcare and social provision in proximity to those concerned.

Currently, there is no willingness to implement a tool common to the health and social sectors to measure loss of independence, even though such a tool is recognized as a prerequisite for the integration of care and services [9]. The tools currently used in France to manage the funding of services that respond to loss of independence appear unreliable and unreproducible. They have not proven to be of metrological value in terms of the degree and nature of the loss of independence, and above all of how this loss of independence evolves over time, as they are insensitive to change [23]. The initial diagnoses and the evaluation of the actions necessary for the management of health policies concerning older people cannot be performed efficiently without a precise multidimensional tool enabling integrated assessment of social and health problems, so as to determine the support needs of the older people. Likewise, none of the latest changes to the health system has involved the use of a single information system for the social and medical sectors, which is a sine qua non of the implementation of an integrated health system [9].

Current funding of healthcare stakeholders in France does not favor the implementation of quality integrated care. Depending on their job and workplace (private, hospital), health professionals (e.g. doctors, registered nurses) may be salaried employees in healthcare or medical-social establishments or self-employed. Deliberations are underway to develop alternative modes of funding stakeholders. Payments modulated by the quality of care were introduced some years ago for general practitioners and healthcare establishments, in addition to the usual funding per procedure and per activity. The aim is to offer financial incentives to professionals and to establishments as a function of the health results achieved in the population they cater to, measured by clinical indicators. These indicators can be patient-reported outcome measures or patient-reported experience measures even though these latter are still little developed in France in this setting. Funding of the care pathway or of an episode of care – a single flat rate for a defined care pathway involving interventions by stakeholders from different sectors – is currently being reviewed (colectomy for cancer, hip prosthesis…). The feasibility and benefits of such funding in terms of efficacy and effectiveness have yet to be demonstrated, given the difficulties associated with the high degree of fragmentation of the French health system. A few care pathways have just been legally authorized and currently concern only complex care in the hospital sector of specific illnesses in precise conditions (renal insufficiency before dialysis and diabetes). Finally, collective funding to support care that is shared and coordinated between different stakeholders has been developed, notably in the framework of care homes and the CPTS. However, there seems to be a reluctance in France to give the CPTS real collective responsibility for the health of the population of the territory concerned, as is the case, for example, of Accountable Care Organizations in the United States, which are remunerated as a function of the quality and efficiency of their performance for a given patient population [24].

Other innovations are underway in the framework of the Article 51 of the 2018 social security funding law. The aim is to support the experimental implementation of an innovative organization able to override various funding rules applicable to private practice and to hospital or medical-social establishments. This will encourage multiprofessional restructuring of outpatient care, the promotion of interprofessional cooperation and skill sharing, thus expediting the articulation and integration of outpatient care, hospital care, and medical-social management, as well as the use of appropriate tools or digital services. In this experimental approach, healthcare professionals or users propose a new organization for a 4-year period to improve patient pathways, health system efficiency, and access to care. Evaluations will indicate whether or not a bottom-up approach should be generalized to the whole of France. At present, it is not clear how the government can use these experimental schemes to modify the health system other than by the addition of complementary services (e.g. provision in the community of dietary care, preventive care, a clinical pharmacist, etc.), which is unfavorable to an integrated care approach.

The role of users and carers in the roll-out of a policy of integrated care and services seems fundamental, since it is the response to the needs of patients that justifies the integration of care [25]. Kodner and Kyriacou explicitly state this in their definition of integration [7]. In France, the National Authority for Health (Haute Autorité de Santé) made a strong statement in September 2020 by publishing a first recommendation designed to support and encourage the involvement of users in the social, medical-social, and health sectors [26]. The role of users is defined on several levels: involvement in their own care or life plan by taking into account their experiences and preferences; involvement in the management of services and in the evaluation of professional practices with a view to shared building; involvement in the design of innovative solutions or in social and medical-social support. The new organizations (territorial support platforms and planned homes for seniors and their carers) are paying particular attention to users’ needs and preferences in defining their life path [3]. However, The role of users and their relatives in the governance and management of these schemes is yet to be defined. Likewise, the role of users in drawing up health policies is tending to increase via the introduction over the last 20 years of a consultative body (Conférences Régionales de Santé et de l’Autonomie) tasked with identifying the needs of the territory’s population and with helping to evaluate the medical-social needs of people who are losing autonomy. Users’ degree of involvement most often remains that of information provision or even consultation, the aim being to move towards a genuine partnership between users, health professionals, and decision makers [27]. This movement creates conditions favorable for assessment of the impact of more integrated care, even if the setting up of such care remains hypothetical.

The COVID-19 crisis has highlighted the fragmentation of the health system, notably between stakeholders of the for-profit, not-for-profit, and public sectors, but also between the acute care and long-term care sectors and between social and medical services. During the first wave of the COVID-19 epidemic, from March to May 2020, the French government decided on the lockdown of the residents of nursing homes for older people. Visits from residents’ families were banned and general practitioners had great difficulty gaining admission to care for their patients, because of the risk of infection, particularly when the nursing home was the site of an outbreak. Usually on their own initiative, mobile geriatric teams (generally intra-hospital) were organized to offer help and support to nursing homes [28,29]. The health authorities quickly set up geriatric COVID-19 hotlines to provide support to the medical and care teams of nursing homes in terms of the clinical management of residents, but also organizationally, and in terms of reassurance regarding management of the epidemic. Because of fragmentation between the health and social systems, home care services remain isolated, despite some local support initiatives (notably via the organization of web conferences for home care professionals and health, geriatrics, and infectious diseases experts, for dissemination of information, questions and answers, experience sharing) [28]. Lastly, the condition of some patients with chronic illnesses worsened because of numerous deferrals of hospital admissions during lockdown [30,31]. In the absence of delegation of responsibilities between healthcare establishments, certain public hospitals may not have referred these patients to private establishments able to receive them because of a fear of diverting patients from the public to the private sector.

In the aftermath of the COVID-19 lockdown, a new health reform was implemented in France in September 2020. This plan, “le Ségur de la santé” (the Ministry of Health being on the Avenue de Ségur in Paris), provides for the investment of 19 billion€ in health. Apart from measures to increase the number of health professionals and to upgrade hospital salaries, this plan emphasizes the development of integrated private/public medical-social care provision for older people. The proposed measures include perpetuation of the COVID-19 lockdown measure of having hospital doctors on-call to nursing homes, the structuring in each territory of direct unplanned hospital admissions for older people, so as to avoid unwarranted admissions to emergency departments, the deployment of specialized mobile teams to the residences of older people, and increased on-call duties for night nurses in nursing homes.

## Conclusion

Over the last decade, efforts have been made to overcome the fragmentation of services for vulnerable people, through an inter-sectoral and multilevel approach, and many attempts have been made both systemically and organizationally to foster the coordination of care and social and health services [5,6]. More recently, there has been a willingness to develop the integration of care, but progress has been limited. If we use Kodner and Kyriacou’s definition [7], we see that while the degree of connectedness has probably increased somewhat (albeit with the persistent major problem of lack of an information system), we are still far from alignment, more so than in 2010, because of fragmentation of the institutions of governance and multiplication of schemes. Collaboration can sometimes be facilitated by improved connectedness, but there is still a lack of shared understanding of the problems, which need to be measured in terms of the care and services shared between various stakeholders. The degree of involvement of providers seems to have been quite high over the last 10 years, as witnessed by the number of initiatives, though the involvement of funders and administrative bodies remains extremely complex because of a failure to make integrated care a political priority. The increasing inclusion of users and of their experience in the assessment of public policies probably offers today the greatest leverage in implementing the hoped-for changes to the French health system.

The French healthcare system in 2020 is probably more fragmented than it was in 2010, despite probable progress in the collaboration and coordination of stakeholders. Numerous hopes formulated 10 years ago have, in the end, not had a tangible impact. Breaks in continuity of national leadership (3 presidents with different political agendas in 10 years, never mind governmental differences) have likely played a large part. Also, many unknowns remain in the practical application of recent and ongoing reforms. The beginning of the “health reform” by the French government following the COVID-19 crisis provides an opportunity to capitalize on this progress and to implement “integrated care,” which is only possible with strong and continuous national leadership, governance, and change management, including a better vision of dedicated funding.

## Lessons learned

- Integration can recede despite stakeholders’ involvement to foster coordination.
- Increasing consideration for patients’ and their carers’ experience may facilitate the elaboration and adaptation of integration policies.
- Several barriers have been identified: multiplication of schemes and programs with no shared tools to measure dependence, no systematic system outcomes to support an evidence-based evaluation, and no integrated and transparent governance and funding of health policies for older people.
- Strong and continuous national leadership is needed to effect change management and to make integrated care a political priority.

## Footnotes

The meaning of the acronym was originally “home for autonomy and integrated care for people suffering from Alzheimer’s disease”: in French “Maison pour l’Autonomie et l’Intégration des malades d’Alzheimer”; the meaning of the acronym was modified in 2011 to “method of action for integration of health and social care in the field of autonomy.”

In France, there are 101 departments grouped into 18 regions and we use “territorial councils” for the English translation of “conseils départementaux.”
